# Determination of lactate value

**DOI:** 10.1590/S1516-31802012000200011

**Published:** 2012-04-03

**Authors:** Viroj Wiwanitkit

**Affiliations:** I MD, PhD. Professor, Wiwanitkit House, Bangkhae, Bangkok, Thailand.; II MD. Attending physician in the Intensive Care Unit, Discipline of Anesthesiology, Pain and Intensive Care, Universidade Federal de São Paulo - Escola Paulista de Medicina (Unifesp-EPM), São Paulo, Brazil.; III MD, MSc. Coordinator of the Intensive Care Unit, Discipline of Anesthesiology, Pain and Intensive Care, Universidade Federal de São Paulo - Escola Paulista de Medicina (Unifesp-EPM), São Paulo, Brazil.; IV MD. Coordinator of the Intensive Care Unit, Discipline of Anesthesiology, Pain and Intensive Care, Universidade Federal de São Paulo - Escola Paulista de Medicina (Unifesp-EPM), São Paulo, Brazil.; V MD, PhD. Adjunct Professor, Discipline of Anesthesiology, Pain and Intensive Care, Universidade Federal de São Paulo - Escola Paulista de Medicina (Unifesp-EPM), São Paulo, Brazil.

Dear Editor,

I read a recently published paper on determination of lactate values with great interest.^1^ It was concluded that “Lcen, and not Lper, can replace Lart with good correlation and clinical agreement. Lper tends to overestimate Lart, thus leading to unnecessary therapeutic interventions”.^1^ There are some concerns regarding this work. First, it has to be clarified that the lactate levels determined at each site are not the same. The values from arterial, venous and capillary blood cannot be interchanged with each another. Second, the quality control for the analytical technique should be mentioned. In general practice, a point-of-care testing tool might be used for capillary blood samples, while a standard clinical chemistry analyzer might be used for arterial and venous blood samples. This may simply result in differences.
