# Supplementary material for: Determination of lactate value
Source: Sao Paulo Med J. 2012 Apr 3;130(2):130–1. doi: 10.1590/S1516-31802012000200011 (PMC10896559; doi:10.1590/S1516-31802012000200011)
Supplement: Related texts

# Reply: RESPONSE TO LETTER TO THE EDITOR

**DOI:** 10.1590/S1516-31802012000200012

**Authors:** Ana Paula Metran Nascente, Murillo Assunção, Carla Janaina Guedes, Flávio Geraldo Rezende Freitas, Bruno Franco Mazza, Miriam Jackiu, Flávia Ribeiro Machado

We really appreciate your interest in our study published in the last issue of Sao Paulo Medical Journal. We would like to thank you for your comments.

We would like to clarify that the aim of our work was exactly to test whether the lactate levels in arterial and venous blood could replace each other. Our results did show that venous peripheral lactate, drawn by venipuncture and not from capillary blood, cannot replace arterial lactate. However, the concordance between central venous and arterial levels was reasonable. This suggests that, in clinical practice, both can be used.

In our hospital, there is no capillary lactate analysis at the bedside. This analysis would have provided us with new and interesting data. However, the rationale presented in the introduction of our paper gives rise to our belief that the capillary sample would be influenced by many factors relating to limb perfusion at the sample collection site. The capillary lactate would reflect the perfusion and metabolism of the compartment drained by that capillary blood (the local perfusion), and not the systemic perfusion.

In the methods section of our study, we presented the technical analysis method used on our samples. We used colorimetric enzymatic assays for all samples. It is important to mention the sensitivity of our technique, with a minimum limit of 1 mg/dl (0.1 mmol/l), and its good specificity. Using this technique, we observed that there was no interference with regard to ascorbic acid values not higher than 30 mg/dl, conjugated and non-conjugated bilirubin not higher than 60 mg/dl, hemoglobin not higher than 1000 mg/dl and triglycerides not higher than 2000 mg/dl. Comparison between the methods available for this same analysis showed a good correlation: r = 0.999. Therefore, we do not believe that technical problems could explain our results.
